# The positive psychology of challenge: Towards interdisciplinary studies of activities and processes involving challenges

**DOI:** 10.3389/fpsyg.2022.1090069

**Published:** 2023-01-13

**Authors:** Keisuke Horikoshi

**Affiliations:** Independent Researcher, Tokyo, Japan

**Keywords:** challenge, well-being, positive psychology, interdisciplinary, integration, challengership, challengership studies

## Abstract

Activities and processes involving challenges are a natural part of life for most people and are highlighted in times of rapid change and global issues. This article argues that more studies around activities and processes involving challenges should be conducted with a focus on the concept of challenge in the context of well-being and optimal functioning. The concept of challenge is important because it is explicitly embedded in many major themes of positive psychology and can be a key concept in creating perspectives and frameworks to connect and integrate multiple elements in positive psychology to promote advancements in the field. Studying activities and processes involving challenges is also important from the perspective of dialectically integrating the positive and negative elements encompassed in the concept of challenge. The article also proposes to label activities and processes involving challenges as “challengership” and that an interdisciplinary area to study “challengership” (named “challengership studies”) should be created, which can collaborate with positive psychology for mutual development. The positive psychology of challenge/challengership is likely to provide opportunities for further advancement of positive psychology by creating more integrated knowledge of how to flourish when faced with challenges individually and collectively. The knowledge created in these areas can also be applied to education, coaching, and training at schools and organizations to meet the needs of the times, where skills of challengership should be considered trainable.

## 1. Introduction

For most people, activities and processes involving challenges, including seeking, identifying, taking, embracing, avoiding, persevering, and overcoming them, are naturally part of life, as they have been throughout the history of humanity. In dictionaries, a challenge is described as “a new or difficult task or situation that tests somebody’s ability and skill” (Oxford Learner’s Dictionaries, n.d.) and as something that can be interpreted as an opportunity (APA Dictionary of Psychology, n.d.). Scholars have studied activities and processes involving the concept of challenge in various disciplines, including psychology. Societal needs in our time of rapid change and global challenges have also highlighted the importance of activities and processes involving challenges concerning well-being.

## 2. Importance of studying activities and processes involving challenges

Although scholars of positive psychology (PP) have suggested the importance of including and integrating challenges in the framework of well-being research (Boniwell, 2012; Lomas and Ivtzan, 2016; Wong, 2016b; Wong et al., 2022), PP studies have tended not to construct integrated perspectives that focus on the concept of challenge. I argue that more studies on activities and processes involving challenges should be conducted with the aim of building integrated, interdisciplinary knowledge that is useful for humanity to flourish in spite of challenges.

### 2.1. The concept of challenge is important in that it is explicitly and extensively embedded in the major themes of PP

From the perspective of PP, with an emphasis on the major themes, the concept of challenge is a critically important element for well-being and optimal functioning because it is explicitly and extensively embedded in the theories, models, and definitions of well-being, flow, intrinsic motivation, curiosity, mindset, learning, stress coping, mental toughness, and posttraumatic growth, among others.

To illustrate this point, examples of 10 important themes encompassed in PP are briefly reviewed in the context of the concept of challenge. (1) Regarding flow and well-being, one of the main conditions for achieving flow is that challenges and skills balance at high levels (Csikszentmihalyi, 2003), and flow is regarded as a primary form of engagement, which is one of the five pillars of well-being in the PERMA model (Seligman, 2011). Dodge et al. (2012, p. 230) related challenges to well-being by defining the latter as “the balance point between an individual’s resource pool and the challenges faced.” In connection with these studies, peak experiences (Maslow, 1959) were also associated with challenges in some studies (e.g., McDonald et al., 2009; Harung, 2012). (2) Regarding intrinsic motivation, Ryan and Deci (2000, p. 56) explained that an intrinsically motivated person is “moved to act for the fun or challenge entailed rather than because of external prods, pressures, or rewards.” Amabile et al. (1994) conceptualized intrinsic motivation in the Work Preference Inventory consisting of two factors: challenge and enjoyment. (3) Regarding curiosity, Kashdan and Silvia (2009, p. 368) defined curiosity as “the recognition, pursuit, and intense desire to explore novel, challenging, and uncertain events.” It should be noted that curiosity is incorporated into the concept of mindfulness (Bishop et al., 2004; Lau et al., 2006; Jazaieri and Shapiro, 2017), which can be then linked to the concept of challenge. (4) Regarding mindset and learning, Dweck and Yeager (2019, p. 482) called the mindset theory “a theory of challenge-seeking and resilience.” Bjork and Bjork (2020) claimed that the existence of challenges and difficulties at an appropriate level is effective for long-term learning. In a study of motor learning, Guadagnoli and Lee (2004) claimed that there is an optimal challenge point for learning. (5) Stress coping models incorporate the concept of challenge. For example, studies have suggested that challenge is more effective than threat for stress appraisal in a transactional model (Lazarus and Folkman, 1984) and as a motivational state in a biopsychosocial model (Blascovich and Mendes, 2000) of stress coping. (6) Mental toughness was conceptualized as a combination of four elements: challenge, confidence, commitment, and control (Clough and Strycharczyk, 2012). Similarly, hardiness has been conceptualized as a combination of three elements: commitment, control, and challenge (Maddi, 2006). (7) Tedeschi and Calhoun (2004) incorporated a high level of challenge into the model of posttraumatic growth. (8) Regarding goal setting, studies indicated that goal setting that incorporates appropriate challenges is effective in realizing higher performance (Locke et al., 1981; Latham and Locke, 1991). (9) Other than these studies, certain character strengths, such as hope, bravery, and persistence, as well as the concepts of resilience and grit, assume some kind of challenges, difficulties, and adversities in which these concepts and qualities play a positive role toward flourishing (Reivich and Shatté, 2002; Snyder, 2002; Peterson and Seligman, 2004; Duckworth et al., 2007). For instance, “I have overcome setbacks to conquer an important challenge.” is one of the 12 items on the Grit Scale (Duckworth et al., 2007, p. 1090). (10) Furthermore, in positive organizational psychology (Donaldson and Ko, 2010), many of the abovementioned themes incorporating the concept of challenge in PP have been applied to studies at the organizational level and in coaching. Examples include applications of flow, growth mindset, mental toughness, character strengths (including curiosity), resilience, and goal setting to organizations and coaching (e.g., Csikszentmihalyi, 2003; Donaldson and Ko, 2010; Murphy and Dweck, 2010; Green and Palmer, 2018; Canning et al., 2020).

In most of the above examples, the concept of challenge does not appear in the primary themes but is explicitly linked to them within respective theories, models, and definitions. Because these important themes are linked to well-being and optimal functioning, the concept of challenge is extensively linked to these conditions as well.

The above examples also indicate that many themes and theories of PP cannot exist without the concept of challenge and that the existence of challenge is one of the pillars of realizing well-being and optimal functioning in some themes. While they indicate the importance of the concept of challenge in PP, it is frequently used without definition to define and theorize other concepts in the above examples. Despite its importance, conceptual analyses as well as systematic perspectives and frameworks to integrate these major themes with a focus on the concept of challenge are missing and therefore need to be developed.

To clarify the arguments further, it is worth describing what a challenge is from a broad integrative perspective, which is based on how the construct of challenge is described in the above examples. Because the examples cover broad themes, they describe multiple aspects encompassed in the construct of challenge. In particular, three interconnected aspects are repeatedly emphasized: (1) challenges are described as difficult, new, or complex (e.g., Csikszentmihalyi, 1990; Latham and Locke, 1991; Amabile et al., 1994; Tedeschi and Calhoun, 2004; Kashdan and Silvia, 2009; Dweck and Yeager, 2019; Bjork and Bjork, 2020); (2) challenges are compared to skills or resources (e.g., Lazarus and Folkman, 1984; Csikszentmihalyi, 2003; Tedeschi and Calhoun, 2004; Dodge et al., 2012) that may be put to the test or eventually developed by pushing own limits; (3) challenges may be interpreted as and eventually transformed into opportunities, including for action, learning, growth, or developing skills and resources (e.g., Lazarus and Folkman, 1984; Amabile et al., 1994; Clough and Strycharczyk, 2012; Nakamura and Csikszentmihalyi, 2014) that are associated with adaptation in some studies (e.g., Lazarus and Folkman, 1984; Tedeschi and Calhoun, 2004). These aspects are mostly concordant with the definition of challenge in some of the major dictionaries (APA Dictionary of Psychology, n.d.; Oxford Learner’s Dictionaries, n.d.). Integrating these aspects can preliminarily define a challenge as a situation, task, or problem that is difficult, new, or complex and presents the possibility of testing skills or resources and being interpreted as or transformed into an opportunity. Although this preliminary working definition is based only on a limited number of examples and should be further examined in more comprehensive studies, its strength lies in it being able to accommodate the essences of the interconnected aspects of the challenges that are studied separately in the above examples.

### 2.2. The concept of challenge is important from the perspectives of second wave and third wave PP

Studies of activities and processes involving challenges are important from the perspective of the so-called second wave PP (SWPP), which emphasizes the dialectical aspect of well-being and the integration of positive and negative elements of life (Wong, 2011; Lomas and Ivtzan, 2016). Activities and processes involving challenges tend to mix positive and negative elements for most people, therefore making their examination an important pursuit from the perspective of SWPP.

The concept of challenge is closely related to that of suffering, another important concept in the context of SWPP (e.g., Bueno-Gómez, 2017; Wong et al., 2021). For example, in some studies, the concept of challenge is used to represent situations involving suffering (e.g., Lomas and Ivtzan, 2016; Wong et al., 2022). Studies of mature happiness and the CasMac model aim to promote flourishing amidst suffering, challenges, and adversity (Wong and Bowers, 2018). Challenges that are too high compared to skills are associated with anxiety in the flow model (Csikszentmihalyi, 1990), and anxiety can cause suffering (Bueno-Gómez, 2017). Studies of flow also indicate that some people in solitary ordeals (e.g., survival crises and captivity) transformed their extreme experiences into flow by seeking appropriate challenges despite their suffering (Logan, 1985; Csikszentmihalyi, 1990). Other examples of the relationship between suffering and challenge include the models of stress coping and posttraumatic growth, incorporating the concept of challenge (Lazarus and Folkman, 1984; Blascovich and Mendes, 2000; Tedeschi and Calhoun, 2004) because suffering (as an umbrella term) can encompass the concepts of stress and trauma (Wong et al., 2021). These examples suggest that there is an essential intersection between studies on suffering and those on activities and processes involving challenges in PP. Therefore, studies on suffering in PP should incorporate and integrate studies of activities and processes involving challenges for further development.

Studies of activities and processes involving challenges are also important from the perspective of the so-called third wave PP (TWPP), which emphasizes the broadening of scope and methods (Lomas et al., 2021), including the adoption of an interdisciplinary approach. In this context, real-world challenges and problems tend to neglect the boundaries of disciplines as well as those among individuals (e.g., Repko and Szostak, 2020; The University of British Columbia, n.d.). Therefore, studies on activities and processes involving challenges require an interdisciplinary approach.

Studies in multiple disciplines other than psychology have also incorporated the concept of challenge in the contexts of well-being and optimal functioning. The following are some examples of this: (1) Many studies on leadership and management have incorporated the concept of challenge in their theories and models, where leadership shapes activities and processes involving challenges, and vice versa. For example, the concept of challenge is incorporated in the conceptualization of challenge-driven leadership (Ancona and Gregersen, 2018) and adaptive challenge (Heifetz et al., 2009). The Big Hairy Audacious Goal as part of leadership (Collins and Lazier, 2020) and secure base leadership (Kohlrieser et al., 2012) are conceptualized to promote the pursuit of challenges. (2) In an example of a study in philosophy, Irvine (2019) integrated the knowledge of Stoic philosophy and psychology and argued that practicing Stoicism is beneficial in dealing with difficulties, adversities, and challenges. (3) Other examples include drama. For instance, improvisation training can be effective in enabling people to take on new challenges because it allows people to train the performance (without preparation) of new actions with confidence in front of unfamiliar audiences, to deal with failure, and to tame the fear related to failure (Madson, 2005). These examples indicate the importance of studying activities and processes involving challenges using an interdisciplinary approach concerning the TWPP.

### 2.3. The concept of challenge is relevant in our time

In the context of the current needs of the society, the concept of challenge is relevant in our time of rapid changes and global challenges, including the COVID-19 pandemic. For example, during the pandemic, the question of how to embrace challenges and flourish is conceptually considered more relevant for many people than the simpler question of how to flourish. This reframing of the question to explicitly include and integrate challenges in the framework of well-being research has been recommended by many PP scholars (Boniwell, 2012; Lomas and Ivtzan, 2016; Wong, 2016b; Wong et al., 2022). Because the framing of questions shapes the quality of answers (Gregersen, 2018), this reframing is critical to living optimally with challenges for people. Based on these considerations, conducting more studies on activities and processes involving challenges in relation to well-being is necessary to meet the demands of the times and to contribute to broad societal needs.

### 2.4. Challenge as a key concept to integrate multiple elements in PP

One line of persistent criticism of PP is that its concepts and theories are fragmented without sufficient integration (e.g., Cowen and Kilmer, 2002; Boniwell, 2012; Wissing, 2022). On this issue, studies on activities and processes involving challenges are likely to offer an important new perspective to connect and integrate some (if not all) of the major themes of PP structured around the concept of challenge. For example, as mentioned earlier, many important themes in PP are directly related to the concept of challenge. While the world is traditionally viewed through the separate conceptual windows that these respective themes construct, studies of activities and processes involving challenges can aim to create new integrative perspectives and frameworks by positioning the concept of challenge at the center, which changes the viewpoints of existing studies.

In the pursuit of such integrative perspectives, one potential focal point may be the concept of an optimal challenge or a challenge at an optimal level that realizes well-being and optimal functioning. One rationale for this perspective is that some studies conceptualize challenge (or difficulty) with its degree or level, assuming that there is an optimal degree or level of challenge (Csikszentmihalyi, 2003; Guadagnoli and Lee, 2004; Dodge et al., 2012; Bjork and Bjork, 2020) rather than as a binary conceptualization. Based on these considerations, one possible direction may be to clarify the optimal levels of challenge in the respective concepts and theories of PP and seek a framework to connect, integrate, and organize them.

Another advantage of studying activities and processes involving challenges is the possible coexistence and integration of the well-being of self and contributions to others. Taking on challenges at optimal levels can be beneficial not only to the well-being of the self but also to others, because such challenges are often difficult tasks or problems that others may want to avoid. For example, creating flow by embracing high-level challenges at work can be beneficial to the well-being of the self and contribution to others (Csikszentmihalyi, 2003). At the awareness level, studies of flow have also indicated a loss of ego in the flow state (Logan, 1985; Csikszentmihalyi, 1990). In another instance, leadership driven by large, inspiring challenges (Ancona and Gregersen, 2018) can be beneficial to well-being and contributions to others. Regardless of whether an individual’s focus is more on the interests of self or others, the consequences of the coexistence of the well-being of self and contributions to others in these examples may be linked to or compared with those driven by self-transcendence (Wong, 2016a). In line with this perspective, flow, peak experiences, and mindfulness, which are categorized as self-transcendent experiences (Yaden et al., 2017), are, respectively, linked with challenges, as reviewed earlier.

## 3. Discussion

As reviewed, the concept of challenge is already embedded in many of the major themes and theories of PP without sufficient integration and is also suited for the dialectical aspect of well-being to integrate the positive and negative elements of life emphasized in SWPP. Given these unique characteristics, the concept of challenge is well-situated and should be studied further to create perspectives and frameworks in which these themes and theories linked with challenges are connected and integrated.

One of the future research directions is to conduct conceptual analyses of challenges in psychology, as well as in related disciplines such as management and philosophy. Specifically, conceptual analyses of challenges may include the relationship between challenges and well-being, optimal functioning, learning, creativity, adaptation, suffering, leadership, entrepreneurship, and coaching, among others. For example, the definition of entrepreneurship as “the pursuit of opportunity beyond the resources you currently control” (Stevenson, 2004, p. 3) may be compared with that of challenge, as the latter also involves the elements of opportunity and resources.

### 3.1. Creating interdisciplinary studies of activities and processes involving challenges in collaboration with PP: “Challengership studies”

For further conceptualization and analyses, I propose to label the activities and processes involving challenges as “challengership.” From a cross-cultural perspective (Berry et al., 2011; Lomas, 2015, 2021), concepts in non-English languages that (at least partially) denote activities and processes involving challenges, such as *chōsen* (挑戦) in Japanese, should also be included under the concept of challengership as an umbrella term.

The preceding section focused on the importance of studies on challengership in relation to PP. In this section, I propose to conceptualize an interdisciplinary area of study of challengership as “challengership studies” (CS) from a wider perspective, including individual and collective levels, independent of the contexts of PP and psychology. The rationale of this formulation is that challengership should be studied using an interdisciplinary approach, as mentioned earlier, and is not necessarily limited to studies of well-being and psychology. Considering the complexity of the world, most people cannot deny the interdisciplinary nature of challenges/challengership. CS and PP intersect to a considerable extent, which can be called the PP of challenge/challengership. Examples of high-level research questions posited at this intersection may include: (1) What is an optimal challenge/challengership? (2) What are the factors that enhance or hinder optimal challengership, individually or collectively? (3) How can we assess and measure challengership? (4) How can we enhance the individual/collective skills of challengership? In pursuit of these important questions, the PP of challenge/challengership can be a base on which CS and PP can collaborate for mutual development to advance well-being and optimal functioning of humanity. Furthermore, knowledge created in these areas can also be applied to education, coaching with a focus on challengership (challengership coaching), and training at schools and organizations in collaboration with positive education and leadership education to meet the needs of the times, where challengership skills should be considered trainable.

In summary, this paper argues that further studies on challengership should be conducted from an integrative perspective. I also propose that an interdisciplinary area to study challengership should be created, which can collaborate with PP for mutual development. The PP of challenge/challengership is likely to provide opportunities for future advancement of PP.

## Data availability statement

The original contributions presented in the study are included in the article/supplementary material, further inquiries can be directed to the corresponding author.

## Author contributions

The author confirms being the sole contributor of this work and has approved it for publication.

## Conflict of interest

The author declares that the research was conducted in the absence of any commercial or financial relationships that could be construed as a potential conflict of interest.

## Publisher’s note

All claims expressed in this article are solely those of the authors and do not necessarily represent those of their affiliated organizations, or those of the publisher, the editors and the reviewers. Any product that may be evaluated in this article, or claim that may be made by its manufacturer, is not guaranteed or endorsed by the publisher.
